# Association of Edentulism with Various Chronic Diseases in Mexican Elders 60+ Years: Results of a Population-Based Survey

**DOI:** 10.3390/healthcare9040404

**Published:** 2021-04-01

**Authors:** Alejandro José Casanova-Rosado, Juan Fernando Casanova-Rosado, Mirna Minaya-Sánchez, José Luís Robles-Minaya, Juan Alejandro Casanova-Sarmiento, María de Lourdes Márquez-Corona, América Patricia Pontigo-Loyola, Horacio Isla-Granillo, Mariana Mora-Acosta, Sonia Márquez-Rodríguez, Carlo Eduardo Medina-Solís, Gerardo Maupomé

**Affiliations:** 1 School of Dentistry, Autonomous University of Campeche, Campeche 24039, Mexico; ajcasano@uacam.mx (A.J.C.-R.); miminaya@uacam.mx (M.M.-S.); jlroblesm17@gmail.com (J.L.R.-M.); juancasanova1192@gmail.com (J.A.C.-S.); 2 Academic Area of Dentistry, Health Sciences Institute, Autonomous University of Hidalgo State, Pachuca 42160, Mexico; lmarquez@uaeh.edu.mx (M.d.L.M.-C.); americap@uaeh.edu.mx (A.P.P.-L.); hislasg@uaeh.edu.mx (H.I.-G.); mtramarianamoraa@hotmail.com (M.M.-A.); cdsoniamr@hotmail.com (S.M.-R.); 3 Advanced Studies and Research Center in Dentistry “Dr. Keisaburo Miyata”, School of Dentistry, Autonomous University of State of Mexico, Toluca 50000, Mexico; 4 Richard M. Fairbanks School of Public Health, Indiana University/Purdue University, Indianapolis, IN 46202, USA; gmaupome@iu.edu; 5 Indiana University Network Science Institute, Bloomington, IN 47408, USA

**Keywords:** oral health, edentulism, chronic diseases, mental disorders, elders, Mexico

## Abstract

Objective: To determine the association of edentulism with different chronic diseases and mental disorders in Mexicans aged 60 years and over. Material and Methods: A cross-sectional study was carried out using data from the World Health Survey for Mexico, in a probabilistic, multi-stage cluster sampling framework. Data for self-report of chronic diseases (diabetes, arthritis, angina pectoris and asthma), mental disorders (depression and schizophrenia) and edentulism were analyzed. Edentulism data were available for 20 of the 32 States of Mexico. Statistical analysis was performed in Stata 14.0 using the svy module for complex sampling (Complex nature under which individuals are sampled). Results: In total 4213 subjects were included, representing a population of 7,576,057 individuals. Mean age was 70.13 ± 7.82 years (range 60 to 98); 56.2% were women. Chronic diseases’ prevalence and mental disorders prevalence were as follows: diabetes 15.0% (N = 1,132,693); arthritis 13.2% (N = 1,001,667); depression 5.5% (N = 414,912); angina pectoris 4.5% (344,315); asthma 3.6% (N = 269,287); and schizophrenia 2.2% (N = 16,988). The prevalence of edentulism was 26.3%, which pertained to 1,993,463 people aged 60 years and over. Angina in women aged 60 to 69 years (*p* < 0.05) and depression in men aged 70 years and over (*p* < 0.0001) were associated with higher prevalence of edentulism. Conclusions: There was generally sparse association between edentulism on chronic diseases and mental disorders included in the study, except for women aged 60 to 69 years for angina, and in men aged 70 and over, for depression. Although our findings are misaligned with previous reports, longitudinal studies are required to test causal and temporal relationships between edentulism with chronic diseases and mental disorders.

## 1. Introduction

Around the world, oral diseases such as dental caries in both dentitions, periodontitis and severe tooth loss are the main oral diseases/conditions [1,2,3]. These represent a public health problem since they have a high prevalence and incidence, and have a significant burden of the disease; they increased dramatically between 1990 and 2015 [1,2,3,4,5]. Unfortunately, it has been reported that oral health has not improved in the last three decades, and oral conditions continue to be a major challenge for health systems in many countries [2]. The number of people with untreated oral conditions is estimated to have increased from 2.5 billion in 1990 to 3.5 billion in 2015 [2]. Similarly, in Mexico, dental caries on primary [6,7,8,9,10] and permanent [8,9,10,11,12,13] dentitions as well as periodontal diseases [14,15,16,17] are presented as challenges to public health. In addition to this, a large percentage of the Mexican population has considerable unmet treatment needs; various studies indicate that they are concentrated in populations with the worst socioeconomic conditions [6,7,8,9,10,11,12,13,14,15,16,17,18,19]. This situation generates a significant disease and economic burden for families and for the Mexican health system [9,10].

Tooth loss, partial or total, is a reflection of the history of dental diseases and treatments that people have undergone throughout their lives. This condition is modified by the attitudes of patients and the clinical decisions of dentists, the dentist-patient relationship, the availability and accessibility of dental services, as well as the treatment philosophies prevailing at the time of dental care delivery [3]. Tooth loss effects in terms of pain, suffering, functional deterioration and decreased quality of life are considerable and expensive. Losing multiple teeth has negative implications at the systemic level for chronic diseases [20,21]. For example, it has been associated to hypertension [22], diabetes [23], peripheral arterial disease [24], cardiovascular and brain disease [25], heart failure, stroke, and death [26], angina [27], overweight and obesity [28], renal disease [29], chronic obstructive pulmonary disease [30], dementia [31], depression [32], cognitive impairment [33], certain types of cancer such as liver cancer [34] and pancreas [35], risk of oral, upper gastrointestinal, pulmonary and pancreatic cancer [36,37] as well as with the presence of multimorbidity (+1 chronic disease) [38].

Population aging in Mexico is one of the most important challenges the country is facing. In a short time, the elderly population will increase relative to the other age groups. People over 65 years of age represented 6.3% in 2010 and 10.4% in 2015. However, by 2050, they are expected to represent 22.5% [39]. As the population of older adults in Mexico increases, interest in diseases likely to be associated with old age also grows. Age is a strong predictor for both tooth loss and edentulism, as well as for chronic diseases and mental disorders. From an epidemiological point of view, between 60% and 80% of the elderly population have at least one chronic disease. Therefore, poor oral health, aging and chronic diseases together represent some of the greatest challenges for health systems because they are highly prevalent in older adults and costly [40]. 

People are now living longer and the impact of poor oral health on the quality of life of older adults is an important public health issue. Efforts need to be strengthened in low- and middle-income countries where periodontal diseases and caries are often “solved” by dental extraction instead of dental conservation. Edentulism is a “final marker of disease burden for oral health” and an important indicator of dental caries and periodontal disease sequels [41,42]. While the prevalence of complete edentulism has reduced over the last decade, tooth loss remains a significant disease worldwide, mainly among the elderly population. However, complete edentulism prevalence varies from country to country and from region to region [42]. Edentulism is a highly prevalent condition globally, with an overall age- and sex-standardized prevalence of 7.6% ranging from as low as 1.4% in Bangladesh and Myanmar up to 15.2% in Brazil. Global prevalence figures among those <50 and ≥50 years were 2.8% and 14.0%, respectively. Age-sex standardized prevalence of edentulism was highest in middle-income countries (MICs) (10.9%), followed by high-income countries (HICs) (8.6%), and low-income countries (LICs) (4.4%). The overall prevalence of edentulism in individuals aged <50 years was 2.8% (LICs 1.6%; MICs 4.3%; HICs 3.5%) with the highest prevalence observed in Zimbabwe (14.5%), Nambia (13.2%), and South Africa (8.2%) [43]. 

Oral infections are believed to increase the risk of systemic disease [44,45]. Oral invasive pathogens appear to induce a systemic inflammatory response through mediators released by the cardiovascular system and liver, increasing the risk of developing systemic infections [44]. The precise relationship between chronic diseases and mental disorders with oral diseases has not been fully clarified, and several theories have been proposed. A possible biological mechanism that links periodontal disease, tooth loss and edentulism, with chronic diseases is the local and systemic inflammation (inflammaging) [45] due to endothelial dysfunction are involved, in addition to microvascular and macrovascular damage [46]. People with chronic systemic diseases/conditions also exhibit lowered immune systems [47,48]. Oral bacteria promote platelet aggregation, a key event in the development of thrombosis, in addition to worsening of atheromas when exposed to periodontal pathogens [49,50,51,52]. Periodontal disease is associated with higher levels of acute phase proteins, plasma antibody levels, coagulation factor, total white blood cell count, neutrophils, C reactive protein (CRP), and cytokines such as IFN-γ (interferon-gamma), TNF-α (Tumor Necrosis Factor-Alpha), IL (Interleukin)-1β, IL-2 and IL-6. [45,49,52]. Tooth loss is associated with non-invasive measures of atherosclerosis, such as thickening of the carotid wall, stenosis, and the presence of carotid plaque [20,49,53]. Taken in aggregate, such evidence may constitute an aggregate of chronic oral infection implicated with various chronic diseases [20,22,23,24,25,26,28,29,34,35,36,37,38,54,55,56], cognitive impairment and other mental issues [20,31,32,33] and death [21,26]. Based on this background, we set out to test the hypothesis that edentulism is associated with chronic diseases and mental disorders in the elderly. The objective of the present study was to determine the strength of association of edentulism with a limited array of chronic diseases and mental disorders in Mexican individuals aged 60 years and over.

## 2. Materials and Methods

### 2.1. Study Design, Population and Sample

This cross-sectional study is a secondary analysis of the National Performance Evaluation Survey (ENED), which was part of the World Health Organization (WHO) Global Health Survey project. Survey methods have been described elsewhere [57] including some oral health results [58,59]. The original data collection instrument was provided by the WHO. For operational reasons, those people living in collective residential dwellings were excluded from the target population. The ENED sample design was probabilistic, multi-stage, stratified and by conglomerates. Three strata were considered: (a) Cities or metropolitan areas (locations with >100,000 inhabitants); (b) urban settings (locations from 2500 to 99,999 inhabitants), and (c) rural areas (locations with fewer than 2500 inhabitants). A sample size of approximately 1243 households was determined per state. The sample size for each stratum was designed proportionally to the number of inhabitants within the stratum to allow representation of both urban and rural areas. The complete WHS questionnaire was not administered to all states; the dental part of the survey was available only for 20 of the 32 states of Mexico (24,159 households). For this analysis, only adults aged 60 years and over were included, leading to a sample of 4213 people.

### 2.2. Variables and Data Collection

The questionnaires were administered by trained personnel at home. We included in the analysis conditions such as edentulism and five chronic systemic diseases and two mental disorders. The questions used to calculate the prevalence of chronic diseases and mental disorders were: Have you ever been diagnosed with arthritis (a disease of the joints)?, Has a doctor or other health professional ever told you that you have angina pectoris (heart disease)?, Have you ever been diagnosed with asthma (an allergic respiratory disease)?, Has a doctor or other health professional ever told you that you suffer from depression?, Have you ever been diagnosed with schizophrenia or psychosis?, Have you ever been diagnosed with diabetes (high blood sugar)? The independent variable was edentulism (absence of all-natural teeth in the mouth, collected through the question: Are you missing all your natural teeth? The variables age (0 = 60–69, 1 = 70 and over) and sex (0 = male, 1 = female) were also included in the stratified analysis.

### 2.3. Statistic Analysis

Due to the design used in the survey sampling, the svy module for complex sampling (Complex nature under which individuals are sampled) of the Stata 14.0^®^ statistical package was used. First, a univariate analysis was carried out, reporting summary measures as appropriate. In the bivariate analysis, the X^2^ test was used. Pearson’s chi-square statistic was corrected by using Rao and Scott’s second-order correction and converted to an F statistic [60]. Since the bivariate analysis did not show an association between the events studied and edentulism, no multivariate models were reported.

### 2.4. Ethical Statement 

Since public databases were used, the approval of the ethics and research committee was not required for this specific sub-analysis. The main study complied with the research and ethics guidelines established by the Helsinki principles and regulations in place for health research in Mexico.

## 3. Results

A total of 4213 participants were included, representing a population of 7,576,057 individuals. All data presented are weighted. Descriptive results are shown in Table 1. Mean age was 70.13 ± 7.82 years (range 60 to 98). Women represented 56.2%. Chronic diseases and mental disorders prevalence were: diabetes 15% (N = 1,132,693); arthritis 13.2% (N = 1,001,667); depression 5.5% (N = 414,912); angina pectoris 4.5% (344,315); asthma 3.6% (N = 269,287); and schizophrenia 2.2% (N = 16,988). The prevalence of multimorbidity was 7.6% (N = 572,659). Prevalence of complete edentulism was 26.3%, which represents 1,993,463 people aged 60 years and over.

Table 2 shows the results of the crude logistic regression analyses; the association with edentulism was significant for none of the chronic diseases and mental disorders. Analyses stratified by age and sex are shown in Table 3, Table 4 and Table 5: with the exception of angina in women aged 60 to 69 years (*p* < 0.05) and depression in men aged 70 years and over (*p* < 0.0001), no statistically significant differences were observed in edentulism through chronic diseases.

## 4. Discussion

The present study aimed to determine the association of edentulism with different chronic diseases and mental disorders in Mexican individuals aged 60 years and over. We observed that 1 in 4 (26.3%) adults were edentulous, but this feature was for the most part not significantly associated with chronic diseases and mental disorders. Chronic conditions often arise and develop in parallel with other diseases. Co-occurrence of chronic conditions and dental conditions have been reported in the literature [54]. It is not clear whether this is true causation or simply an association between oral infections and some other systemic conditions [55]. Methodologically speaking, a major consideration is that multiple studies have been observational and cross-sectional, confirming statistical associations [22,23,27,28,29] but not causal relationships between chronic conditions and edentulism. When large scale cohort studies (accruing more confidence in the suggestion of a causal relationship but not reaching the level of certainty) are examined, the association is maintained [24,26,30,31]. In a meta-analysis of cohort studies [25] it was a concluded that design and quality of studies—together with the number of cases and participants—support the association between tooth loss and both cardiovascular disease and cerebrovascular incidents. Other meta-analyses give credence to a link between death risk [21], obesity [28], dementia [61], depression [32], Alzheimer’s disease [62], asthma [63], different types of cancer [64,65] and metabolic syndrome [66] and oral health indicators. Oral diseases/infections as a risk factor for the development of various systemic conditions is a topic that has been widely investigated and debated. Although most of the evidence for this association consistently supports this notion, the need for further studies is apparent. In general, studies with larger populations and better designs corroborate the association of dental conditions with systemic conditions.

People with chronic conditions are more likely to have untreated dental disease, which can in turn lead to tooth loss [67]. The association under evaluation was not confirmed by our findings. One possible reason for not finding an association between chronic diseases and edentulism is that the present study included adults over 60 years of age: people in poorer health may be less than 60 years of age, thereby self-selecting them out of the pool of participants (Sixty years of age, and the selection of chronic conditions, were determined by the methods and priorities set in the national survey and derived from health policy guidelines in Mexico).

Among the study’s greatest strengths is the nationwide representativeness of the sample and strong methodological design. One of the limitations of the study is its cross-sectional design, which leads to temporal ambiguity through measuring cause and effect at the same time. The cross-sectional design precludes detecting trends in the disease patterns under evaluation. Self-report is an efficient and accepted approach to collecting data about salient health conditions; it is a standard feature of the Global Health Survey. However, there are risks of recall bias (perhaps more pronounced in older individuals) or misrepresentation of conditions believed to be present, but not based on a formal medical diagnosis. Finally, in the present study only edentulism was measured: data on the exact number of missing teeth were not measured for each individual—possibly diminishing precision of the variable [34]. 

Based on our findings, further research is necessary to examine data through a more finely grained set of strategies: e.g., adding other chronic diseases to the limited array included in the national survey; establishing levels of severity of disease, together with measures of how well controlled some diseases are (such as glycemic control in diabetics); incorporating other Latin American population groups besides Mexicans; and disaggregating the block of 60+ age group in various levels. Finally, longitudinal designs would be required to fully quantify causality between chronic diseases, mental disorders, and tooth loss and, separately, with edentulism (complete tooth loss).

## 5. Conclusions

Few associations were observed between edentulism and the chronic diseases and mental disorders included in the study. In the stratified analysis, edentulism was associated only with angina in women aged 60 to 69 years, and with depression in men aged 70 and over. More research is needed to clarify the association of edentulism with selected chronic diseases and mental disorders, and to characterize mechanisms for tooth loss.

## Figures and Tables

**Table 1:** Variables from Mexican subjects aged 60 and older.

Variable	Frequency	N	% Weighted
Age			
60–69	2227	4,194,885	55.4
70 and older	1986	3,381,172	44.6
Gender			
Female	2364	4,259,135	56.2
Male	1849	3,316,922	43.8
Edentulism			
No	3053	5,582,594	73.7
Yes	1160	1,993,463	26.3
Diabetes			
No	3640	6,443,364	85.0
Yes	573	1,132,693	15.0
Arthritis			
No	3709	6,574,390	86.8
Yes	504	1,001,667	13.2
Depression			
No	4011	7,161,145	94.5
Yes	202	414,912	5.5
Angina pectoris			
No	3990	7,231,742	95.5
Yes	223	344,315	4.5
Asthma			
No	4062	7,306,770	96.4
Yes	151	269,287	3.6
Schizophrenia			
No	4199	7,559,069	99.8
Yes	14	16,988	2.2
Multimorbidity			
No	3922	7,003,398	92.4
Yes	291	572,659	7.6

**Table 2:** Crude estimates (Odds ratio= OR, 95% confidence interval=95% CI) of the different chronic diseases, mental disorders and multimorbidity (2 or more chronic diseases at the same time) and edentulism in Mexican older adults.

	**Diabetes**	**Value *p***	**Arthritis**	**Value *p***	**Depression**	**Value *p***
Edentulism						
No	1 *		1 *		1 *	
Yes	0.94 (0.64–1.37)	0.767	1.23 (0.90–1.68)	0.175	1.22 (0.83–1.78)	0.297
	**Angina**	**Value *p***	**Asthma**	**Value *p***	**Schizophrenia**	**Value *p***
Edentulism						
No	1 *		1 *		1 *	
Yes	1.30 (0.77–2.19)	0.317	0.68 (0.35–1.30)	0.243	1.81 (0.44–7.43)	0.408
	**At least one**	**Value *p***	**Multimorbidity**	**Value *p***		
Edentulism						
No	1 *		1 *			
Yes	1.10 (0.84–1.43)	0.463	1.09 (0.80–1.50)	0.560		

At least one = Refers to any chronic disease or mental disorders present among those included in the study. * Reference category.

**Table 3:** Distribution of edentulism due to diabetes and arthritis stratified by age group and sex (estimated population N = 7,576,057).

Variables	Diabetes		Arthritis	
**Females**	**No**	**Yes**	**No**	**Yes**
Edentulism (60–69)				
No	1,489,447 (82.0)	327,475 (18.0)	1,570,035 (86.4)	246,887 (13.6)
Yes	450,061 (87.0)	67,488 (13.0)	435,048 (84.1)	82,501 (15.9)
X^2^ test	*p* = 0.1675		*p* = 0.4519	
Edentulism (70 and older)				
No	1,001,633 (85.7)	166,973 (14.3)	951,499 (81.4)	217,107 (18.6)
Yes	637,792 (84.4)	118,266 (15.6)	607,583 (80.4)	148,475 (19.6)
X^2^ test	*p* = 0.7314		*p* = 0.7498	
**Males**	**No**	**Yes**	**No**	**Yes**
Edentulism (60–69)				
No	1,398,600 (87.6)	198,512 (12.4)	1,467,854 (91.9)	129,258 (8.1)
Yes	214,843 (81.6)	48,459 (18.4)	236,482 (89.8)	26,820 (10.2)
X^2^ test	*p* = 0.2983		*p* = 0.6737	
Edentulism (70 and older)				
No	847,797 (84.8)	152,157 (15.2)	892,262 (89.2)	107,692 (10.8)
Yes	403,191 (88.3)	53,363 (11.7)	413,627 (90.6)	42,927 (9.4)
X^2^ test	*p* = 0.4677		*p* = 0.7077	

**Table 4:** Distribution of edentulism due to depression and angina stratified by age group and sex (estimated population N = 7,576,057).

Variables	Depression		Angina	
**Females**	**No**	**Yes**	**No**	**Yes**
Edentulism (60–69)				
No	1,714,925 (94.4)	101,997 (5.6)	1,762,043 (97.0)	54,879 (3.0)
Yes	463,873 (89.6)	53,676 (10.4)	478,668 (92.5)	38,881 (7.5)
X^2^ test	*p* = 0.0743		*p* = 0.0499	
Edentulism (70 and older)				
No	1,051,633 (90.0)	116,973 (10.0)	1,102,916 (94.4)	65,690 (5.6)
Yes	715,033 (94.6)	41,025 (5.4)	708,310 (93.7)	47,748 (6.3)
X^2^ test	*p* = 0.0768		*p* = 0.8001	
**Males**	**No**	**Yes**	**No**	**Yes**
Edentulism (60–69)				
No	1,529,155 (95.7)	67,957 (4.3)	1,546,560 (96.8)	50,552 (3.2)
Yes	251,548 (95.5)	11,754 (4.5)	259,392 (98.5)	3910 (1.5)
X^2^ test	*p* = 0.9388		*p* = 0.2960	
Edentulism (70 and older)				
No	997,062 (99.7)	2892 (0.03)	935,229 (93.5)	64,725 (6.5)
Yes	437,916 (95.9)	18,638 (4.1)	438,624 (96.1)	17,930 (3.9)
X^2^ test	*p* = 0.0000		*p* = 0.2651	

**Table 5:** Distribution of edentulism due to asthma and schizophrenia stratified by age group and sex (estimated population N = 7,576,057).

Variables	Asthma		Schizophrenia	
**Females**	**No**	**Yes**	**No**	**Yes**
Edentulismo (60–69)				
No	1,728,845 (95.2)	88,077 (4.8)	1,812,928 (99.8)	3994 (0.2)
Yes	507,448 (98.0)	10,101 (2.0)	514,678 (99.4)	2871 (0.6)
X^2^ test	*p* = 0.1611		*p* = 0.3980	
Edentulism (70 and older)				
No	1,107,494 (94.8)	61,112 (5.2)	1,167,502 (99.9)	1104 (0.1)
Yes	727,897 (96.3)	28,161 (3.7)	755,017 (99.9)	1041 (0.1)
X^2^ test	*p* = 0.4259		*p* = 0.2747	
**Males**	**No**	**Yes**	**No**	**Yes**
Edentulism (60–69)				
No	1,561,253 (97.8)	35,859 (2.2)	1,595,130 (99.9)	1982 (0.1)
Yes	260,033 (98.8)	3269 (1.2)	263,098 (99.9)	204 (0.1)
X^2^ test	*p* = 0.3461		*p* = 0.7095	
Edentulism (70 and older)				
No	968,950 (96.9)	31,004 (3.3)	996,710 (99.7)	3244 (0.3)
Yes	444,850 (97.4)	11,704 (2.6)	454,006 (99.4)	2548 (0.6)
X^2^ test	*p* = 0.7353		*p* = 0.6960	

## Data Availability

The data sets generated during and analyzed during the current study are available from the corresponding author on reasonable request.
